# Expression of Concern: Proxalutamide Reduces the Rate of Hospitalization for COVID-19 Male Outpatients: A Randomized Double-Blinded Placebo-Controlled Trial

**DOI:** 10.3389/fmed.2021.831449

**Published:** 2022-01-17

**Authors:** Frontiers Editorial Office

With this notice, Frontiers states its awareness of serious complaints regarding this article. This expression of concern has been posted while Frontiers investigates the allegations. The situation will be updated as soon as the investigation is complete.

